# Tunneling nanotubes: an alternate route for propagation of the bystander effect following oncolytic viral infection

**DOI:** 10.1038/mto.2016.29

**Published:** 2016-12-07

**Authors:** Justin Ady, Venugopal Thayanithy, Kelly Mojica, Phillip Wong, Joshua Carson, Prassanna Rao, Yuman Fong, Emil Lou

**Affiliations:** 1 Department of Surgery, Memorial Sloan-Kettering Cancer Center, New York, New York, USA; 2 Department of Medicine, Division of Hematology, Oncology and Transplantation, University of Minnesota, Minneapolis, Minnesota, USA

## Abstract

Tunneling nanotubes (TNTs) are ultrafine, filamentous actin-based cytoplasmic extensions which form spontaneously to connect cells at short and long-range distances. We have previously described long-range intercellular communication via TNTs connecting mesothelioma cells *in vitro* and demonstrated TNTs in intact tumors from patients with mesothelioma. Here, we investigate the ability of TNTs to mediate a viral thymidine kinase based bystander effect after oncolytic viral infection and administration of the nucleoside analog ganciclovir. Using confocal microscopy we assessed the ability of TNTs to propagate enhanced green fluorescent protein (eGFP), which is encoded by the herpes simplex virus NV1066, from infected to uninfected recipient cells. Using time-lapse imaging, we observed eGFP expressed in infected cells being transferred via TNTs to noninfected cells; additionally, increasing fluorescent activity in recipient cells indicated cell-to-cell transmission of the eGFP-expressing NV1066 virus had also occurred. TNTs mediated cell death as a form of direct cell-to-cell transfer following viral thymidine kinase mediated activation of ganciclovir, inducing a unique long-range form of the bystander effect through transmission of activated ganciclovir to nonvirus-infected cells. Thus, we provide proof-of-principle demonstration of a previously unknown and alternative mechanism for inducing apoptosis in noninfected recipient cells. The conceptual advance of this work is that TNTs can be harnessed for delivery of oncolytic viruses and of viral thymidine kinase activated drugs to amplify the bystander effect between cancer cells over long distances in stroma-rich tumor microenvironments.

## Introduction

Oncolytic viruses have been investigated for their potential use in cancer therapeutics for decades, culminating with the US Food and Drug Administration’s (FDA) approval of a modified form of oncolytic herpes simplex virus (talimogene laherparepvec) for treatment of human patients with metastatic melanoma.^1^ While progressive generations of novel oncolytic vectors reflect ongoing advances in genetic engineering technology, some of the most basic mechanisms of viral oncolysis remain incompletely understood. The bystander effect is a well-established mechanism by which the effects of oncolytic viruses are propagated to neighboring uninfected cells. This phenomenon effectively amplifies the effects of the virus.^2–4^ Several studies have shown that because of this bystander effect, as few as 10% of all tumor cells need to be transduced with the viral vector to induce complete cell death or tumor regression, even with replication incompetent vectors.^3,5,6^ Currently, the most clearly documented mechanism contributing to the bystander effect involves traffic of lethal agents through gap-junctions between connecting adjacent cells. Studying the bystander effect in ganciclovir (GCV)/viral thymidine kinase (*TK*) gene therapy, Mesnil *et al.* found that GCV monophosphorylated by HSV-TK in an infected cell would travel via gap junctions to effect cell death in uninfected adjacent cells.^5,7–10^ Of note, this mechanism of bystander-oncolysis is entirely dependent upon cell-to-cell contact. However, other groups have demonstrated that some degree of bystander killing can persist even in the absence of gap-junctions.^11^ These data suggest the existence of additional unidentified mechanisms for bystander killing. This is a particularly important concept considering that gap junctions are only able to connect adjacent cells, and thus cannot propagate the bystander effect between distant cells. This context is especially relevant clinically as invasive difficult-to-treat tumors have been shown to contain high proportions of stromal matrix that separate malignant cells, and which have been associated with worse prognosis.^12–19^ The importance of identifying modes of intercellular communication in virology has become magnified by recent studies reporting that over half of HIV virus infections occur through direct cell-to-cell transfer of the virus.^20,21^

The purpose of this study was to investigate the role of tunneling nanotubes (TNTs) as unique intercellular conduits capable of propagating a long-range form of the bystander effect following oncolytic viral infection of cancer cells. TNTs are long, fine, nonadherent actin-based cytoplasmic extensions capable of forming direct connections between cells at both close and distant proximity.^22^ We have shown that TNTs exist in mesothelioma and lung cancer cell lines, in primary cancer cells from effusions, and in a variety of intact resected tumors from human patients.^23–27^ We previously demonstrated that TNTs connecting mesothelioma cells are capable of transferring a variety of types of cellular cargo, including proteins, mitochondria, lipophilic components from the cytosol, and Golgi vesicles.^24^ Notably, TNTs connecting GFP- or RFP-expressing mesothelioma cells facilitated intercellular exchange of these proteins. In addition, TNTs or similar intercellular bridges have previously been shown to transmit infectious agents, such as prions,^28^ viruses, and viral components.^29–35^ Furthermore, recently Roberts *et al.* have demonstrated intercellular transmission of Influenza A virus connected by TNTs.^36^ What is not known is the potential role of TNTs on drug delivery. Using the above information and our prior studies on TNTs as background, for this study, we wanted to further investigate whether TNTs could be harnessed to facilitate therapeutic drug delivery using the bystander effect mediated by an oncolytic viral vector as an *in vitro* model. We investigate the effects on TNTs on the bystander effect induced by oncolytic virus infection. These results provide proof-of-concept data supporting the idea that TNTs can be utilized to amplify the effects of cancer drug and oncolytic viral therapy. This finding presents a conceptual advance of how we can view the bystander effect in the context of a 3-dimensional and heterogeneous tumor matrix, and provides a strong basis for investigation of the effects of TNT formation between cells infected with oncolytic viruses.

## Results

### Virally infected mesothelioma cells form TNTs

**Figure 1:**
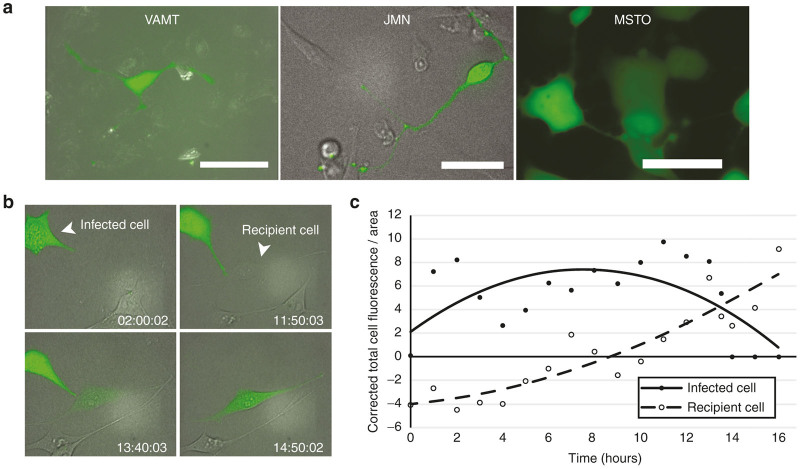
TNTs mediate transfer of eGFP from a NV1066-infected mesothelioma cell to a noninfected mesothelioma cell. (**a**) NV1066-infected VAMT, JMN, and MSTO mesothelioma cells form TNTs following viral infection. Scale bars = 20 μm. (**b**) Time-lapse microscopy of a JMN mesothelioma cell infected with eGFP-expressing NV1066 forming a TNT that mediates eGFP transfer to a noninfected cell. This transfer took place over ~10–12 hours. (**c**) Quantification of eGFP expression in the infected (donor) and recipient cells from panel **b** as over time, reported using corrected total cell fluorescence per area (CTCF/Area). eGFP, enhanced green fluorescence protein; TNT, tunneling nanotube.

We selected three cell lines of varying histologic subtypes of malignant mesothelioma (MSTO-211H, VAMT, and JMN) for this study, as we had previously demonstrated effective *in vitro* formation of TNTs in this form of cancer.^24,37,38^ To determine whether mesothelioma cells infected with HSV are capable of transferring virally encoded proteins between cells via TNTs, we infected mesothelioma cells from each cell line separately with NV1066, a mutant HSV strain that encodes the readily-expressed viral enhanced green fluorescence protein (eGFP) transgene.^39–42^ After consistent formation of TNTs between malignant mesothelioma cells was observed, NV1066 was added. Live cells were examined using confocal fluorescent imaging 12–36 hours after addition of virus. GFP was present diffusely in both the cell body and along the length of TNTs connecting two or more cells (Figure 1a,b; also Supplementary Movies S1–S4). This experiment confirmed that mesothelioma cells infected with an oncolytic virus form TNTs. NV1066-infected cells formed TNTs beyond 18 hours before succumbing to viral oncolysis; this finding indicates that viral oncolytic infection did not preclude TNT formation as the life-cycle of the herpes simplex type-1 virus (HSV-1) virion particle is 18 hours. This is further supported in Supplementary Figure S1, in which we demonstrate that infection of MSTO-211H cells with NV1066 did not preclude the formation of TNTs. In fact, at 48 hours postviral infection there were significantly more TNTs per cell when compared with controls (Supplementary Figure S1).

### TNTs mediate intercellular transfer of oncolytic virus NV1066 and of virus-encoded eGFP

Next we cocultured virally infected cells with noninfected cells and performed time-lapse confocal imaging to capture TNT formation between these two populations. Using time-lapse fluorescent microscopy, we identified direct intercellular transfer of eGFP between cells along TNTs for as long as 16 hours after initial infection (Figure 1b and Supplementary Movies S1–S4). Similar results were observed with all virally infected cell lines. In one particularly striking example, a GFP-expressing virally infected cell formed a direct TNT connection to a noninfected cell, to which it subsequently transferred the virus, which was tracked by its green fluorescence GFP product (Figure 1b and Supplementary Movie S1). We assessed the shift in GFP from donor to recipient cell by calculating the corrected total cell fluorescence per area as we have previously described.^26^ The corrected total cell fluorescence /area for the initially infected cell peaked and then decreased, while the corrected total cell fluorescence /area in the recipient cell receiving viral eGFP increased steadily (Figure 1c). These data demonstrate that virally encoded proteins, such as eGFP, can be expressed and replicated in infected cells and then be transmitted between cells connected by TNTs, including from infected to noninfected cells. Importantly, the fact that eGFP expression continued to increase beyond the termination of the TNT connection also strongly suggested that the replication-competent virus itself was also transferred to the recipient cell. This was further supported by the set of time-lapse images shown in Supplementary Movie S2, in which a GFP-expressing cell forms extensive and curved TNT to a noninfected target cell which undergoes cell death following connection and transmission of GFP/virus.

### TNTs can facilitate amplification of the bystander effect by transferring viral TK-activated GCV from infected to noninfected cells

The most prominent suicide gene used in preliminary studies of oncolytic viral therapy has been the *TK* gene of HSV-1.^3,4,7^ Expression of this gene sensitizes virally-transduced cells to selective drugs; in the case of HSV-1 TK, the most commonly used drug is GCV, a nucleoside analog which is 10 times more effective than acyclovir (ACV) in inducing apoptosis.^7^ The effectiveness of suicide gene therapy rests on the principle of the so-called “bystander effect” in which cancer cells transduced by the virus cause activation of the prodrug. This activated prodrug is then transferred to surrounding cells resulting in both the death of the infected cells as well as surrounding uninfected cells.^5^

To determine whether TNTs mediate the bystander effect, we used a modified transwell assay to investigate TNT-mediated intercellular transfer of GCV between virally infected cells and noninfected cells. It has been well-established that gap junctions are a primary cellular mediator of the bystander effect, for cells in immediate proximity and connected via these connexin-lined channels. As gap junctions and TNTs are not mutually exclusive, but rather play complementary roles spatially for cells in close proximity or located at a distance away from each other, with communication most efficiently facilitated by gap junctions and TNTs, respectively.

**Figure 2:**
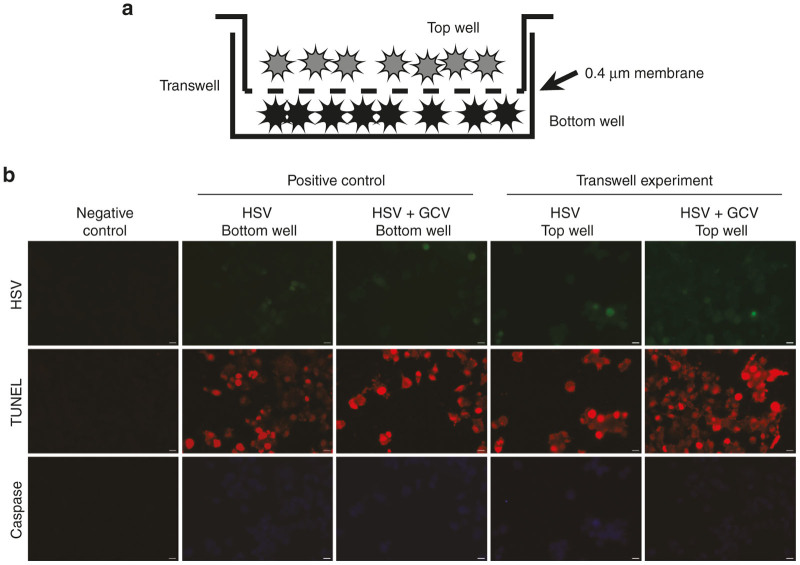
Use of a modified transwell assay to assess a TNT-mediated bystander effect following oncolytic viral infection. (**a**) Schematic drawing of the modified transwell assay/Boyden chamber used to assess TNT-mediated transfer. (**b**) Representative fluorescence micrographs of TUNEL staining of apoptotic MSTO-211H mesothelioma cells in the bottom well of the transwell assay. Green fluorescence shows virus-encoded eGFP expression. Red fluorescence shows TUNEL-positive nuclei. Blue fluorescence indicates expression of Caspase-3. eGFP, enhanced green fluorescence protein; TNT, tunneling nanotube; TUNEL, terminal deoxynucleotidyl transferase nick-end labeling.

**Figure 3:**
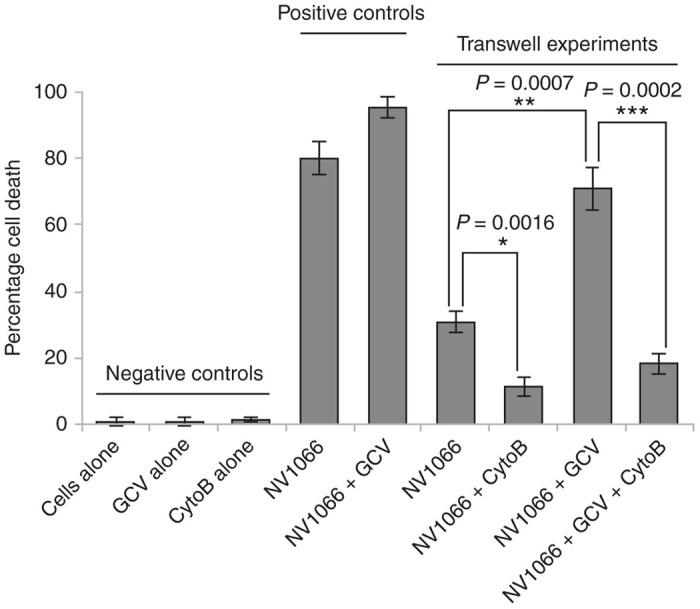
TNTs enhance the bystander effect via transfer of virus and ganciclovir to uninfected mesothelioma cells. Quantitative analysis of the number of TUNEL-positive cells in the bottom well of the transwell assay at 48 hours. Data were analyzed using the student’s *t*-test, and are graphed as the mean ± SD. *n* = 3 for each group. Scale bars = 200 µm. SD, standard deviation; TNT, tunneling nanotube; TUNEL, terminal deoxynucleotidyl transferase nick-end labeling.

For the purposes of this study, we postulated that TNTs provide an additional or alternate route by which a long-range form of the bystander effect may take place. Thus in order to examine the long-distance effect of TNTs independent of gap junctions, a polyester filter with the smallest commercially available pore size (400 nm = 0.4 µm) was used to separate the two cell populations (depicted in Figure 2a). This approach permits TNT formation while abrogating the ability of cells to form connexin-based gap junctions, as compared with adjoining cells in proximity in open culture.^27,43–45^ The filter also serves as an effective physical barrier to reduce diffusion and trafficking of exosomes or microvesicles by as much as 85%^46,47^; for further details, please see “Methods” section. Mesothelioma cells were added to the top well of the transwell assay after infection with NV1066 with or without addition of GCV. Apoptosis was measured using terminal deoxynucleotidyl transferase nick-end labeling (TUNEL) assay in the bottom chamber to assess whether TNT-mediated transfer of NV1066 and GCV affected the bystander effect (Figure 2b). Quantification of TUNEL-positive cells at 48 hours indicated that addition of NV1066 to the top chamber resulted in 33% of the initially uninfected cells in the bottom chamber dying by 48 hours (Figure 3). The addition of GCV to virally infected cells in the top chamber significantly increased apoptosis in recipient cells in the bottom chamber, from 33–71%, producing a 2.3-fold increase in cell kill attributed to TNT transfer of viral TK-activated GCV (*P* = 0.007) (Figure 3).

To ensure that the difference in apoptosis was not due to transfer of virus through the filter pores into the bottom well, we assessed addition of NV1066 directly to the bottom well (positive control) and found that 80% of cells in the bottom well were apoptotic as measured by TUNEL staining. We noted that even with direct addition of virus, the cell death was not 100% even after 48 hours. Additionally, adding GCV with NV1066 (positive control) to the bottom well significantly increased apoptosis further to 95%; notably, while the percentage cell death increased by 10–15%, it still did not induce 100% cell death. No effect was observed when GCV alone was added to the bottom well or when cells were cultured alone in the bottom well without viral infection or addition of GCV (negative controls; Figure 3). Together, these data strongly suggest that NV1066-infected donor cells in the top chamber caused viral TK-induced activation of GCV, which then was transferred to recipient cells in the bottom well via TNTs.

### Pharmacologic inhibition of TNTs as a negative control: use of the actin destabilizing agent Cytochalasin B

Additional measures taken to ensure this bystander effect did not occur via exosomal transfer of activated GCV included use of serum-free medium and incubation of cells at 4°C for 4 hours prior to start of the invasion assay, both measures known to suppress exosomal secretion.^48^ One negative control for the experiment involved pharmacologic inhibition of TNT formation.

To date there is no known pharmacologic agent that exclusively targets TNTs. Thus to accomplish this part of the experiment demonstrating that inhibiting TNT formation prevented the bystander effect, we treated cells in separate assays with 400 nmol/l cytochalasin B (CytoB), a well-studied actin-destabilizing agent known to prevent formation of TNTs.^49^ Previous work had demonstrated that actin-depolymerizing agents such as CytoB and D, Latrunculin A, azide, colchicines, and tubulin inhibitors block TNT formation or traffic along TNTs.^34,49–51^ CytoB has been well-studied and used extensively for negative controls in *in vitro* studies of TNTs, due to its ability to destabilize actin. We used this drug at doses similar to previous studies which demonstrate its ability to prevent formation^49,52^ or disrupt^53^ TNTs. In our assay, CytoB was added at the same time as NV1066 virus; as compared with no drug, the addition of CytoB led to >50% reduction in cell death; the increase in amount of cell death when GCV was also added was minimal, indicating that the role of potential non-TNT transfer of viral TK-activated GCV through the membrane filter was minimal.

Cell killing at 48 hours was significantly decreased when CytoB was added to the top chamber containing NV1066 alone (30 versus 11%; *P* = 0.0016) or both NV1066 and GCV (18 versus 71%; *P* = 0.002; Figure 3). These findings indicate that abrogation of TNTs reduced transfer of activated GCV to noninfected mesothelioma cells. The difference between NV1066 to the top chamber (30% cell death) and addition of NV1066 + GCV (~75%) was striking and statistically significant (*P* = 0.0007). Taken together, these data support the notion that activated GCV was transferred from the top to the bottom chambers via TNTs.

## Discussion

Our study demonstrates for the first time that TNTs provide an effective and previously undemonstrated alternate route for long-range cellular therapeutic drug delivery through amplification of the bystander effect following oncolytic viral treatment and activation through phosphorylation of a viral TK-activated drug (the nucleoside analog GCV). Using confocal and time-lapse imaging, we observed the presence of TNTs in mesothelioma cells infected with HSV. Our observations demonstrated that there was no significant decrease in the formation of TNTs after infection with HSV when compared with controls. We then demonstrated that TNTs were involved in long-range intercellular transport of NV1066 virus and viral-encoded eGFP. As a result of this finding, we postulate that virally infected cells may also transmit proapoptotic messages to noninfected proximal and distal cells. Previously the paradigm of the bystander effect relied on cells being located in immediate proximity and being linked via gap junctions. Thus our study represents a conceptual advance that would explain, at least in part, how cells are able to transmit these messages over a long-range distance within the complex and heterogeneous three-dimensional tumor matrix.

The *TK* gene of HSV-1 is the most prominent suicide gene used in preliminary studies of oncolytic viral therapy.^3,4,7^ The “bystander effect” results not only in the death of cancer cells transduced by HSV-1 upon activation of the prodrug, but also in the death of surrounding uninfected cells.^5^ The form of TK provided by HSV-1 has a much higher specificity for GCV than does cellular TK kinase; thus, this prodrug is selectively activated, via monophosphorylation, in cells which have been infected with the vector.^5^ Once the initial phosphorylation step has taken place, cellular thymidine kinases are then capable of adding the second and third phosphate groups necessary for complete activation of GCV. This triphosphorylated nucleoside can then be incorporated into DNA during nucleic acid synthesis to induce chain termination, resulting in apoptosis. Additionally, once GCV has been phosphorylated initially, it may be transferred to neighboring uninfected cells by engulfment of apoptotic vesicles or via gap junctions. This monophosphorylated GCV can then be phosphorylated normally by cellular TK, where it causes recipient cells to undergo apoptosis.^5,7^ Several studies have shown that, because of this bystander effect, as few as 10% of all tumor cells can be transduced with a viral vector and still result in complete cell death or tumor regression.^3,5,6^ Our group has previously demonstrated that the NV1066 viral vector can induce apoptosis in neighboring, uninfected cancer cells^54^; in light of the results reported here, it is very plausible that the mechanism for this effect was through direct cell-to-cell communication via TNT connections, and that inhibiting apoptosis may further improve the efficacy of the TNT-mediated bystander effect.

In at least three studies, investigators used the transwell assay with similar specifications (same commercially available experimental set-up, filter with 0.4 µm pores) and approach to assess dependence of cargo transfer on cell-to-cell contact.^55–57^ Positive controls labeled in these and similar studies as “coculture” are similar to our positive control which was labeled as “NV1066,” *i.e.*, effects of direct addition of virus to the bottom well of the plate, without barrier filtration. Thus we modeled our experimental design after these and other similar effective studies. However, we also took further steps to control for the diffusion of exosomes or other soluble factors that could also potentially alter the results. As the primary goal of this study was to provide proof-of-principle that TNTs are capable of mediating the bystander effect, we focused efforts on demonstrating TNT-mediated communication while focusing on the goal of eliminating, or at least drastically reducing, secretion or uptake of exosomes as mediators of intercellular transfer. The use of a membrane filter in transwell assays has been shown to reduce exosomal transfer by as much as 85% (ref. ^48^; also unpublished data from our laboratory, manuscript submitted). We used several additional measures including use of serum-free medium (to reduce serum-based exosomes), and incubation of cells at 4°C for 4 hours (to reduce exosomal secretion). We have separately used confocal imaging to provide visual demonstration of TNTs transiting through the membrane filter pores (manuscript submitted), providing further support for using this approach.

The current paradigm of the bystander effect includes the idea that it depends on cell-to-cell contact.^9^ This contact can be mediated by intercellular gap junctions, which allow for the transfer of small cytotoxic molecules, such as activated GCV, to be exchanged between cells, from those which are TK-positive to those which are TK-negative.^8,9^ The possibility of transfer via the extracellular environment has been previously studied, but was dismissed in light of strong evidence supporting the critical role of gap junctions.^8^ Thus, until now it has been widely assumed that gap junctions alone are responsible for propagation of the bystander effect. However, the transfer of viral gene products via TNTs is not mutually exclusive of the transfer of such products via gap junctions. The impact of other modes of intercellular communication—for example, microvesicles or exosomes—may also impact the initiation and continuation of the bystander effect as mediated by both gap junctions and TNTs. Recently published data from our group indicates that exosomes may in fact work synergistically with mesothelioma cells to stimulate intercellular TNT formation, and subsequently enter and utilize these TNTs as direct conduits for cell-to-cell transport.^26^ It will be imperative to investigate the physiologic impact of TNTs on oncolytic viral infection and on the bystander effect within the tumor-stromal matrix *in vivo*.

## Materials and Methods

### Cell lines and culture medium

The human mesothelioma cell lines MSTO-211H (biphasic histology), JMN, and VAMT (sarcomatoid) (American Type Culture Collection (ATCC), Rockville, MD) were used in this study. These cell lines were selected as they were representative of the various histologic subtypes of mesothelioma, and because we had previously demonstrated reproducible TNT formation in each line. All cell lines were passaged in plasmocin-containing medium (Invivogen, San Diego, CA) and tested negative for mycoplasma contamination. Cell lines were authenticated by the Core Fragment Analysis Facility at Johns Hopkins University using short tandem repeat profiling. All cells were cultured using Roswell Park Memorial Institute (RPMI)-1640 medium supplemented with 100 U/ml penicillin, 100 mg/ml streptomycin, and 10% fetal calf serum (FCS) (Life Technologies; Carlsbad, CA). Cells were tested and confirmed to be negative for mycoplasma infection. Cells were maintained in a 5% CO_2_ humidified incubator at 37°C. To stimulate formation of TNTs, cells were cultured in low-serum, hyperglycemic, acidified medium, as previously described,^24^ using RPMI-1640 supplemented with 2.5% FCS, 50 mmol/l glucose, 1% penicillin-streptomycin, 2% L-glutamine. Drug treatment with GCV (AK Scientific, Union City, CA, Catalog no. 71317) was performed by solubilizing the drug in culture medium.

### Viral vectors

The oncolytic vector NV1066 is a replication-competent, attenuated HSV-1 expressing eGFP; construction of this virus has been described previously.^39^ Briefly, this virus is derived from the wild-type HSV-1 F-strain backbone, with single copy deletions of *ICP-4*, *ICP-0*, and *γ1–34.5* that increase tumor selectivity and decrease virulence.^39,58^ NV1066 contains an eGFP transgene under the control of a constitutively active cytomegalovirus promoter. Infected cells thus express eGFP, which can be detected *in vitro* using fluorescence microscopy. Viral stocks were propagated on Vero cells and tittered by standard plaque assay.

### Identification of TNTs using inverted or confocal microscopy

TNTs were identified as previously described in detail.^22,24,25,27,38,59^ Briefly, these parameters included (i) lack of adherence to the substratum of tissue culture plates, including visualization of TNTs passing over adherent cells; (ii) TNTs connecting two cells or extending from one cell were counted if the width of the extension was estimated to be <1,000 nm; and (iii) a narrow base at the site of extrusion from the plasma membrane. Cellular extensions not clearly consistent with the above parameters were excluded. An Olympus IX70 inverted microscope (Olympus Corporation, Waltham, MA) with 20× objective lens was used to identify TNTs. Representative image of TNTs in stated conditions are as shown and as described in the text and in accompanying figures.

### Quantification of TNTs/cell in NV1066 virus-infected mesothelioma cells

MSTO-211H (MSTO) cells were cultured in conditions favorable to TNT formation (RPMI-1640 medium with 2.5% FCS, 50 mmol/l glucose) as described previously.^24^ 5 × 10^3^ MSTO cells were seeded in 24-well tissue culture plates (Fisher Scientific, Pittsburgh, PA). After 72 hours, cells were infected with NV1066 at a multiplicity of infection (MOI) of 0.1; at 24 and 48 hours postinfection, five random fields of view containing a minimum of 50 cells/HPF were selected using a 20× objective lens on a Nikon Eclipse Ti inverted microscope (Nikon Instruments, Melville, NY). TNTs and cells were quantified; these results were averaged and reported as number of TNTs/cell. A *t*-test was used to determine statistical significance (considered at *P*<0.05).

### GCV treatment

As the prodrug GCV is activated by HSV-1 viral TK, the drug was added to the culture of transfected cells in transwell assays to induce the bystander effect. Cells were first infected with the oncolytic vector NV1066, a mutant strain of HSV-1 that retains the endogenous form of viral TK. Cultures were then treated with GCV (AK Scientific, Catalog no. 71317) by adding low serum, hyperglycemic RPMI supplemented with 1 μg/ml GCV (final concentration) to cell culture at 0 hours of the experiment; Boyden chamber assays were performed up to 48 hours.

### Preparation of cells and addition of virus for Boyden chamber assays

Cells were grown using RPMI-1640 medium supplemented with 10% FCS, 25 mmol/l glucose, 1% penicillin-streptomycin, 2% L-glutamine at 37°C with 5% CO_2_ for 2 days. On day 3, in order to remove cell-derived exosomes/microvesicles and also to avoid contamination from FCS-derived exosomes/microvesicles, culture medium was removed and cells were washed with serum-free mTESR medium three times and cultivated using fresh mTESR medium for 24 hours prior to use. On day 4, cells were first prepared by incubation of flasks at 4°C for 3 hours; this step was performed per prior protocols to decrease cell-derived exosome secretion.^27,48^ Cells were then washed with cold phosphate buffered saline (PBS), harvested by trypsinization and plated in transwell top-chamber inserts (1.5 × 10^5^ cells/well) and incubated for one night prior to viral infection. The next morning, NV-1066 virus was added at 0.1 MOI in 500 μl culture medium and incubated at 37°C for 2 hours to permit adsorption of virus into cells. After 4 hours, cells were washed three times with passage medium and then placed in 37°C for incubation for 48 hours with or without addition of 10 µmol/l GCV.

### CytoB treatment

Inhibition of TNTs was performed by addition of CytoB at a concentration of 400 nmol/l, consistent with previously reported protocols.^49^ CytoB was added at the same time point as virus administration to the samples.

### Assessment of apoptosis/cell kills using TUNEL Assay

Cells were assayed for apoptosis at 24 and 48 hours after NV1066 virus infection by staining with EthD-1 (Life Technologies, Rockville, MD). Cells were washed twice with Dulbecco’s Phosphate-Buffered Saline with calcium and magnesium and incubated with 4 mmol/l EthD-1 for 10 minutes. Stained cells were viewed and imaged using the red channel in fluorescent microscopy (IX70, Olympus). Positive assessment of apoptotic cells was made by detection per standard protocols, as damaged nuclear membranes react positively to EthD-1 staining. Using a 10× objective lens, we counted cells in three randomly selected areas and scored EthD-1-positive cells for apoptosis. Apoptosis was also quantitated using standard TUNEL assay at 24 and 48 hours after virus infection using the Click-iT Assay kit (#C10618) per the manufacturer’s protocol (Life Technologies). Cells were imaged using an Axio-Observer Z1 inverted microscope (Carl Zeiss Microscopy GmBH, Oberkochen, Germany) at 100× and 200× magnifications. Using 10× objective, we counted cells in three randomly selected areas and scored cells reacting positively for TUNEL assay as positive for apoptosis. These three scores were averaged and average numbers were plotted. These values were comparable to the score of the EthD-1 staining. For analysis of apoptosis using TUNEL, results were validated using the student’s *t*-test. Average values were calculated from three independent experiments and plotted as shown; values derived from standard deviation (SD) analysis were plotted as error bars.

### Immunohistochemistry of Caspase-3

To further confirm apoptosis, positivity for caspase-3 expression was tested using affinity-purified mouse monoclonal antibody (Catalog no. 437800, Life Technologies, Rockville, MD) and goat antimouse antibody tagged with Alexa Fluor-405 (Catalog no. A31553, Life Technologies).

Cells were rinsed in PBS and fixed using 3.7% paraformaldehyde solution at 37°C for 30 minutes. Paraformaldehyde was subsequently removed and cells were washed three times with PBS, then permeabilized by incubating in 0.1% Triton X-100 in PBS for 5 minutes at room temperature and washed again three times with PBS. Cells were blocked with blocking buffer, (0.1% Tween-20 in 3% bovine serum albumin (BSA) in PBS for 2 hours). Then cells were incubated with 2 µg/ml of primary antibody affinity-purified mouse monoclonal antibody (#437800, Life Technologies) in PBS containing 1% BSA and 0.1% Tween 20 for 2 hours at 4ºC. The cells were washed three times with PBS containing 0.1% Tween-20 for 10 minutes at room temperature and incubated with 2.0 µg/ml of goat antimouse antibody tagged with Alexa Fluor-405 (#A31553, Life Technologies).

### Time-lapse microscopy

Time-lapse imaging was performed by culturing cells in low serum, hyperglycemic medium as described above in clear-bottomed delta-T culture dishes (Bioptechs, Butler, PA). Live-cell imaging was performed at 24 and 48 hours to assess formation of TNTs. Cells with TNTs were imaged using Differential Interference Contrast (DIC) and a confocal spinning disk microscope (Perkin Elmer UltraView ERS Rapid Confocal Imager, Waltham, MA, Axiovert 200M inverted stand) or Zeiss LSM 5 Live confocal microscope at the Memorial Sloan-Kettering Cancer Center (MSKCC) Molecular Cytology Core Facility.

For experiments assessing transmission of viral vector gene products between cells via TNTs, cells were infected with NV1066 after 48 hours. Approximately 12–36 hours after transfection, cells were visualized by DIC (Perkin Elmer UltraView ERS Rapid Confocal Imager microscope or Zeiss LSM 5 Live confocal microscope). Culture dishes were placed in a humidified chamber with 5% CO_2_ at 37°C for the duration of microscopic evaluation. Time-lapse imaging was performed using a 40× objective lens by taking images every 3–4 minutes for up to 3 hours.

### Study approval

This study was carried out at Memorial Sloan-Kettering Cancer Center and at the University of Minnesota in accordance with protocols approved by the Institutional Biosafety Committees (IBC) at both institutions.

## Author Contributions

E.L. and Y.F. conceived of the project and designed experiments. J.A., V.T., K.M., P.W., and J.C. conducted experiments and acquired data. J.A., V.T., P.R., Y.F., and E.L. analyzed the data. J.A., V.T., Y.F. and E.L. wrote the manuscript. All authors reviewed and edited the manuscript for final approval prior to submission.

## Notes

The authors declared no conflict of interest.

The last two authors contributed equally to this work.

## References

[bib1] Ott, PA and Hodi, FS (2016). Talimogene Laherparepvec for the treatment of advanced melanoma. Clin Cancer Res 22: 3127–3131. 27146699 10.1158/1078-0432.CCR-15-2709

[bib2] Bennett, JJ, Tjuvajev, J, Johnson, P, Doubrovin, M, Akhurst, T, Malholtra, S et al. (2001). Positron emission tomography imaging for herpes virus infection: Implications for oncolytic viral treatments of cancer. Nat Med 7: 859–863. 11433353 10.1038/89991

[bib3] Culver, KW, Ram, Z, Wallbridge, S, Ishii, H, Oldfield, EH and Blaese, RM (1992). *In vivo* gene transfer with retroviral vector-producer cells for treatment of experimental brain tumors. Science 256: 1550–1552. 1317968 10.1126/science.1317968

[bib4] Moolten, FL (1986). Tumor chemosensitivity conferred by inserted herpes thymidine kinase genes: paradigm for a prospective cancer control strategy. Cancer Res 46: 5276–5281. 3019523

[bib5] Freeman, SM, Abboud, CN, Whartenby, KA, Packman, CH, Koeplin, DS, Moolten, FL et al. (1993). The “bystander effect”: tumor regression when a fraction of the tumor mass is genetically modified. Cancer Res 53: 5274–5283. 8221662

[bib6] Nagamachi, Y, Tani, M, Shimizu, K, Yoshida, T and Yokota, J (1999). Suicidal gene therapy for pleural metastasis of lung cancer by liposome-mediated transfer of herpes simplex virus thymidine kinase gene. Cancer Gene Ther 6: 546–553. 10608351 10.1038/sj.cgt.7700080

[bib7] Mesnil, M and Yamasaki, H (2000). Bystander effect in herpes simplex virus-thymidine kinase/ganciclovir cancer gene therapy: role of gap-junctional intercellular communication. Cancer Res 60: 3989–3999. 10945596

[bib8] Bi, WL, Parysek, LM, Warnick, R and Stambrook, PJ (1993). *In vitro* evidence that metabolic cooperation is responsible for the bystander effect observed with HSV tk retroviral gene therapy. Hum Gene Ther 4: 725–731. 8186287 10.1089/hum.1993.4.6-725

[bib9] Yang, L, Chiang, Y, Lenz, HJ, Danenberg, KD, Spears, CP, Gordon, EM et al. (1998). Intercellular communication mediates the bystander effect during herpes simplex thymidine kinase/ganciclovir-based gene therapy of human gastrointestinal tumor cells. Hum Gene Ther 9: 719–728. 9551619 10.1089/hum.1998.9.5-719

[bib10] Dilber, MS, Abedi, MR, Christensson, B, Björkstrand, B, Kidder, GM, Naus, CC et al. (1997). Gap junctions promote the bystander effect of herpes simplex virus thymidine kinase *in vivo*. Cancer Res 57: 1523–1528. 9108455

[bib11] Tanaka, T, Duflot-Dancer, A, Tiraby, M, Piccoli, C, Tiraby, G, Yamasaki, H et al. (2009). Bystander effect from cytosine deaminase and uracil phosphoribosyl transferase genes *in vitro*: a partial contribution of gap junctions. Cancer Lett 282: 43–47. 19342154 10.1016/j.canlet.2009.02.050

[bib12] Maitra, A, Iacobuzio-Donahue, C, Rahman, A, Sohn, TA, Argani, P, Meyer, R et al. (2002). Immunohistochemical validation of a novel epithelial and a novel stromal marker of pancreatic ductal adenocarcinoma identified by global expression microarrays: sea urchin fascin homolog and heat shock protein 47. Am J Clin Pathol 118: 52–59. 12109856 10.1309/3PAM-P5WL-2LV0-R4EG

[bib13] Liang, WS, Craig, DW, Carpten, J, Borad, MJ, Demeure, MJ, Weiss, GJ et al. (2012). Genome-wide characterization of pancreatic adenocarcinoma patients using next generation sequencing. PLoS One 7: e43192. 23071490 10.1371/journal.pone.0043192 PMC3468610

[bib14] Dekker, TJ, van de Velde, CJ, van Pelt, GW, Kroep, JR, Julien, JP, Smit, VT et al. (2013). Prognostic significance of the tumor-stroma ratio: validation study in node-negative premenopausal breast cancer patients from the EORTC perioperative chemotherapy (POP) trial (10854). Breast Cancer Res Treat 139: 371–379. 23709090 10.1007/s10549-013-2571-5

[bib15] de Kruijf, EM, van Nes, JG, van de Velde, CJ, Putter, H, Smit, VT, Liefers, GJ et al. (2011). Tumor-stroma ratio in the primary tumor is a prognostic factor in early breast cancer patients, especially in triple-negative carcinoma patients. Breast Cancer Res Treat 125: 687–696. 20361254 10.1007/s10549-010-0855-6

[bib16] Mesker, WE, Liefers, GJ, Junggeburt, JM, van Pelt, GW, Alberici, P, Kuppen, PJ et al. (2009). Presence of a high amount of stroma and downregulation of SMAD4 predict for worse survival for stage I-II colon cancer patients. Cell Oncol 31: 169–178. 19478385 10.3233/CLO-2009-0478 PMC4618830

[bib17] Wu, Y, Grabsch, H, Ivanova, T, Tan, IB, Murray, J, Ooi, CH et al. (2013). Comprehensive genomic meta-analysis identifies intratumoural stroma as a predictor of survival in patients with gastric cancer. Gut 62: 1100–1111. 22735568 10.1136/gutjnl-2011-301373

[bib18] Song, S, Nones, K, Miller, D, Harliwong, I, Kassahn, KS, Pinese, M et al. (2012). qpure: A tool to estimate tumor cellularity from genome-wide single-nucleotide polymorphism profiles. PLoS One 7: e45835. 23049875 10.1371/journal.pone.0045835 PMC3457972

[bib19] Huijbers, A, Tollenaar, RA, v Pelt, GW, Zeestraten, EC, Dutton, S, McConkey, CC et al. (2013). The proportion of tumor-stroma as a strong prognosticator for stage II and III colon cancer patients: validation in the VICTOR trial. Ann Oncol 24: 179–185. 22865778 10.1093/annonc/mds246

[bib20] Galloway, NL, Doitsh, G, Monroe, KM, Yang, Z, Muñoz-Arias, I, Levy, DN et al. (2015). Cell-to-cell transmission of HIV-1 is required to trigger pyroptotic death of lymphoid-tissue-derived CD4 T cells. Cell Rep 12: 1555–1563. 26321639 10.1016/j.celrep.2015.08.011 PMC4565731

[bib21] Iwami, S, Takeuchi, JS, Nakaoka, S, Mammano, F, Clavel, F, Inaba, H, et al. (2015). Cell-to-cell infection by HIV contributes over half of virus infection. Elife 4. doi: 10.7554/eLife.08150. 10.7554/eLife.08150 PMC4592948 26441404

[bib22] Rustom, A, Saffrich, R, Markovic, I, Walther, P and Gerdes, HH (2004). Nanotubular highways for intercellular organelle transport. Science 303: 1007–1010. 14963329 10.1126/science.1093133

[bib23] Lou, E, Fujisawa, S, Barlas, A, Romin, Y, Manova-Todorova, K, Moore, MA et al. (2012). Tunneling nanotubes: a new paradigm for studying intercellular communication and therapeutics in cancer. Commun Integr Biol 5: 399–403. 23060969 10.4161/cib.20569 PMC3460850

[bib24] Lou, E, Fujisawa, S, Morozov, A, Barlas, A, Romin, Y, Dogan, Y et al. (2012). Tunneling nanotubes provide a unique conduit for intercellular transfer of cellular contents in human malignant pleural mesothelioma. PLoS One 7: e33093. 22427958 10.1371/journal.pone.0033093 PMC3302868

[bib25] Ady, JW, Desir, S, Thayanithy, V, Vogel, RI, Moreira, AL, Downey, RJ et al. (2014). Intercellular communication in malignant pleural mesothelioma: properties of tunneling nanotubes. Front Physiol 5: 400. 25400582 10.3389/fphys.2014.00400 PMC4215694

[bib26] Thayanithy, V, Babatunde, V, Dickson, EL, Wong, P, Oh, S, Ke, X et al. (2014). Tumor exosomes induce tunneling nanotubes in lipid raft-enriched regions of human mesothelioma cells. Exp Cell Res 323: 178–188. 24468420 10.1016/j.yexcr.2014.01.014 PMC4159162

[bib27] Thayanithy, V, Dickson, EL, Steer, C, Subramanian, S and Lou, E (2014). Tumor-stromal cross talk: direct cell-to-cell transfer of oncogenic microRNAs via tunneling nanotubes. Transl Res 164: 359–365. 24929208 10.1016/j.trsl.2014.05.011 PMC4242806

[bib28] Gousset, K, Schiff, E, Langevin, C, Marijanovic, Z, Caputo, A, Browman, DT et al. (2009). Prions hijack tunnelling nanotubes for intercellular spread. Nat Cell Biol 11: 328–336. 19198598 10.1038/ncb1841

[bib29] Sherer, NM and Mothes, W (2008). Cytonemes and tunneling nanotubules in cell-cell communication and viral pathogenesis. Trends Cell Biol 18: 414–420. 18703335 10.1016/j.tcb.2008.07.003 PMC2628975

[bib30] Sherer, NM, Lehmann, MJ, Jimenez-Soto, LF, Horensavitz, C, Pypaert, M and Mothes, W (2007). Retroviruses can establish filopodial bridges for efficient cell-to-cell transmission. Nat Cell Biol 9: 310–315. 17293854 10.1038/ncb1544 PMC2628976

[bib31] Xu, W, Santini, PA, Sullivan, JS, He, B, Shan, M, Ball, SC et al. (2009). HIV-1 evades virus-specific IgG2 and IgA responses by targeting systemic and intestinal B cells via long-range intercellular conduits. Nat Immunol 10: 1008–1017. 19648924 10.1038/ni.1753 PMC2784687

[bib32] Eugenin, EA, Gaskill, PJ and Berman, JW (2009). Tunneling nanotubes (TNT): A potential mechanism for intercellular HIV trafficking. Commun Integr Biol 2: 243–244. 19641744 10.4161/cib.2.3.8165 PMC2717534

[bib33] Eugenin, EA, Gaskill, PJ and Berman, JW (2009). Tunneling nanotubes (TNT) are induced by HIV-infection of macrophages: a potential mechanism for intercellular HIV trafficking. Cell Immunol 254: 142–148. 18835599 10.1016/j.cellimm.2008.08.005 PMC2701345

[bib34] Rudnicka, D, Feldmann, J, Porrot, F, Wietgrefe, S, Guadagnini, S, Prévost, MC et al. (2009). Simultaneous cell-to-cell transmission of human immunodeficiency virus to multiple targets through polysynapses. J Virol 83: 6234–6246. 19369333 10.1128/JVI.00282-09 PMC2687379

[bib35] Sowinski, S, Jolly, C, Berninghausen, O, Purbhoo, MA, Chauveau, A, Köhler, K et al. (2008). Membrane nanotubes physically connect T cells over long distances presenting a novel route for HIV-1 transmission. Nat Cell Biol 10: 211–219. 18193035 10.1038/ncb1682

[bib36] Roberts, KL, Manicassamy, B and Lamb, RA (2015). Influenza A virus uses intercellular connections to spread to neighboring cells. J Virol 89: 1537–1549. 25428869 10.1128/JVI.03306-14 PMC4300760

[bib37] Ady, JW, Desir, S, Thayanithy, V, Vogel, RI, Moreira, AL, Downey, RJ et al. (2014). Intercellular communication in malignant pleural mesothelioma: properties of tunneling nanotubes. Front Physiol 5: 400. 25400582 10.3389/fphys.2014.00400 PMC4215694

[bib38] Thayanithy, V, Babatunde, V, Dickson, EL, Wong, P, Oh, S, Ke, X et al. (2014). Tumor exosomes induce tunneling nanotubes in lipid raft-enriched regions of human mesothelioma cells. Exp Cell Res 323: 178–188. 24468420 10.1016/j.yexcr.2014.01.014 PMC4159162

[bib39] Wong, RJ, Joe, JK, Kim, SH, Shah, JP, Horsburgh, B and Fong, Y (2002). Oncolytic herpesvirus effectively treats murine squamous cell carcinoma and spreads by natural lymphatics to treat sites of lymphatic metastases. Hum Gene Ther 13: 1213–1223. 12133274 10.1089/104303402320138998

[bib40] Adusumilli, PS, Chan, MK, Chun, YS, Hezel, M, Chou, TC, Rusch, VW et al. (2006). Cisplatin-induced GADD34 upregulation potentiates oncolytic viral therapy in the treatment of malignant pleural mesothelioma. Cancer Biol Ther 5: 48–53. 16294031 10.4161/cbt.5.1.2237 PMC1383726

[bib41] Adusumilli, PS, Stiles, BM, Chan, MK, Mullerad, M, Eisenberg, DP, Ben-Porat, L et al. (2006). Imaging and therapy of malignant pleural mesothelioma using replication-competent herpes simplex viruses. J Gene Med 8: 603–615. 16475242 10.1002/jgm.877 PMC1804293

[bib42] Adusumilli, PS, Chan, MK, Hezel, M, Yu, Z, Stiles, BM, Chou, TC et al. (2007). Radiation-induced cellular DNA damage repair response enhances viral gene therapy efficacy in the treatment of malignant pleural mesothelioma. Ann Surg Oncol 14: 258–269. 17080237 10.1245/s10434-006-9127-4

[bib43] Wang, X and Gerdes, HH (2012). Long-distance electrical coupling via tunneling nanotubes. Biochim Biophys Acta 1818: 2082–2086. 21930113 10.1016/j.bbamem.2011.09.002

[bib44] Sherer, NM (2013). Long-distance relationships: do membrane nanotubes regulate cell–cell communication and disease progression? Mol Biol Cell 24: 1095–1098. 23580190 10.1091/mbc.E12-08-0622 PMC3623630

[bib45] Wang, X, Veruki, ML, Bukoreshtliev, NV, Hartveit, E and Gerdes, HH (2010). Animal cells connected by nanotubes can be electrically coupled through interposed gap-junction channels. Proc Natl Acad Sci USA 107: 17194–17199. 20855598 10.1073/pnas.1006785107 PMC2951457

[bib46] Munoz, JL, Bliss, SA, Greco, SJ, Ramkissoon, SH, Ligon, KL and Rameshwar, P (2013). Delivery of functional anti-miR-9 by mesenchymal stem cell-derived exosomes to glioblastoma multiforme cells conferred chemosensitivity. Mol Ther Nucleic Acids 2: e126. 24084846 10.1038/mtna.2013.60 PMC4027430

[bib47] Zomer, A, Vendrig, T, Hopmans, ES, van Eijndhoven, M, Middeldorp, JM and Pegtel, DM (2010). Exosomes: fit to deliver small RNA. Commun Integr Biol 3: 447–450. 21057637 10.4161/cib.3.5.12339 PMC2974077

[bib48] Escrevente, C, Keller, S, Altevogt, P and Costa, J (2011). Interaction and uptake of exosomes by ovarian cancer cells. BMC Cancer 11: 108. 21439085 10.1186/1471-2407-11-108 PMC3072949

[bib49] Bukoreshtliev, NV, Wang, X, Hodneland, E, Gurke, S, Barroso, JF and Gerdes, HH (2009). Selective block of tunneling nanotube (TNT) formation inhibits intercellular organelle transfer between PC12 cells. FEBS Lett 583: 1481–1488. 19345217 10.1016/j.febslet.2009.03.065

[bib50] Jung, S, Park, J-Y, Joo, J-H, Kim, Y-M, and Ha, K-S (2011). Extracellular ultrathin fibers sensitive to intracellular reactive oxygen species: Formation of intercellular membrane bridges. Exp Cell Res 317 (12): 1763–1773. 21356206 10.1016/j.yexcr.2011.02.010

[bib51] Onfelt, B, Nedvetzki, S, Benninger, RK, Purbhoo, MA, Sowinski, S, Hume, AN et al. (2006). Structurally distinct membrane nanotubes between human macrophages support long-distance vesicular traffic or surfing of bacteria. J Immunol 177: 8476–8483. 17142745 10.4049/jimmunol.177.12.8476

[bib52] Yang, Y, Otte, A and Hass, R (2015). Human mesenchymal stroma/stem cells exchange membrane proteins and alter functionality during interaction with different tumor cell lines. Stem Cells Dev 24: 1205–1222. 25525832 10.1089/scd.2014.0413 PMC4425222

[bib53] Vallabhaneni, KC, Haller, H and Dumler, I (2012). Vascular smooth muscle cells initiate proliferation of mesenchymal stem cells by mitochondrial transfer via tunneling nanotubes. Stem Cells Dev 21: 3104–3113. 22676452 10.1089/scd.2011.0691 PMC3495124

[bib54] Stanziale, SF, Petrowsky, H, Adusumilli, PS, Ben-Porat, L, Gonen, M and Fong, Y (2004). Infection with oncolytic herpes simplex virus-1 induces apoptosis in neighboring human cancer cells: a potential target to increase anticancer activity. Clin Cancer Res 10: 3225–3232. 15131064 10.1158/1078-0432.ccr-1083-3

[bib55] Sturlan, S, Sachet, M, Baumann, S, Kuznetsova, I, Spittler, A and Bergmann, M (2009). Influenza a virus induces an immediate cytotoxic activity in all major subsets of peripheral blood mononuclear cells. PLoS One 4: e4122. 19125202 10.1371/journal.pone.0004122 PMC2610492

[bib56] Rechavi, O, Goldstein, I, Vernitsky, H, Rotblat, B and Kloog, Y (2007). Intercellular transfer of oncogenic H-Ras at the immunological synapse. PLoS One 2: e1204. 18030338 10.1371/journal.pone.0001204 PMC2065899

[bib57] Goldwich, A, Prechtel, AT, Mühl-Zürbes, P, Pangratz, NM, Stössel, H, Romani, N et al. (2011). Herpes simplex virus type I (HSV-1) replicates in mature dendritic cells but can only be transferred in a cell-cell contact-dependent manner. J Leukoc Biol 89: 973–979. 21427206 10.1189/jlb.0310180

[bib58] Adusumilli, PS, Eisenberg, DP, Chun, YS, Ryu, KW, Ben-Porat, L, Hendershott, KJ et al. (2005). Virally directed fluorescent imaging improves diagnostic sensitivity in the detection of minimal residual disease after potentially curative cytoreductive surgery. J Gastrointest Surg 9: 1138–46; discussion 1146. 16269385 10.1016/j.gassur.2005.06.029 PMC1783680

[bib59] Abounit, S, Delage, E and Zurzolo, C (2015). Identification and characterization of tunneling nanotubes for intercellular trafficking. Curr Protoc Cell Biol 67: 12.10.1–12.10.21. 26061240 10.1002/0471143030.cb1210s67

