# Supplementary material for: Tunneling nanotubes: an alternate route for propagation of the bystander effect following oncolytic viral infection
Source: Mol Ther Oncolytics. 2016 Dec 7;3:16029–. doi: 10.1038/mto.2016.29 (PMC5142513; doi:10.1038/mto.2016.29)
Supplement: Supplementary Figures and Movies [file mto201629-s1.zip › MTO-00109-T-s05.pdf]

Supplementary Figures and Movies

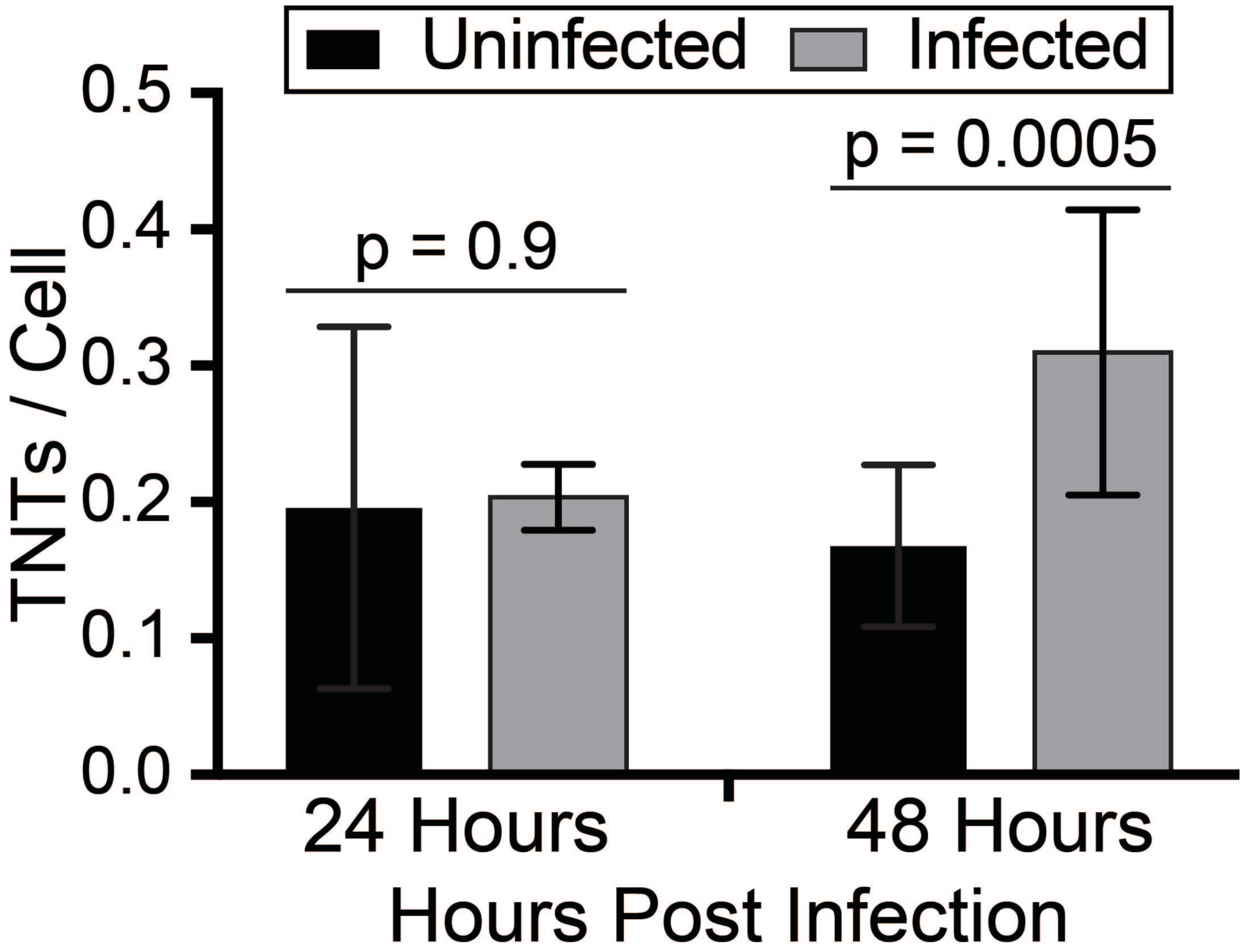

Figure S1. Quantitative analysis of the number of TNTs per cell at both 24 and 48 hours after MSTO-211H cell infection with NV1066. Data were analyzed using student's t-test, and are graphed as the means of ± SD . n=5 for each group. does not prevent the formation of TNTs. In fact at 48 hours post viral infection there were significantly more TNTs per cell when compared to controls

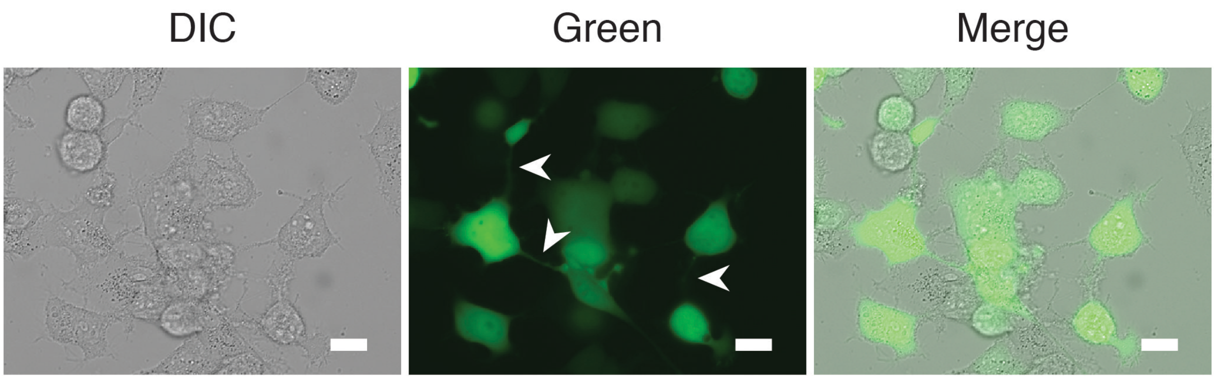

Figure S2. Intercellular transfer of NV1066-expressed eGFP between MSTO mesothelioma cells. DIC and fluorescence images were taken using inverted microscopy at 24 hours following infection; the third image is an overlay produced by merging the first two images. Scale bars: 20 μm.

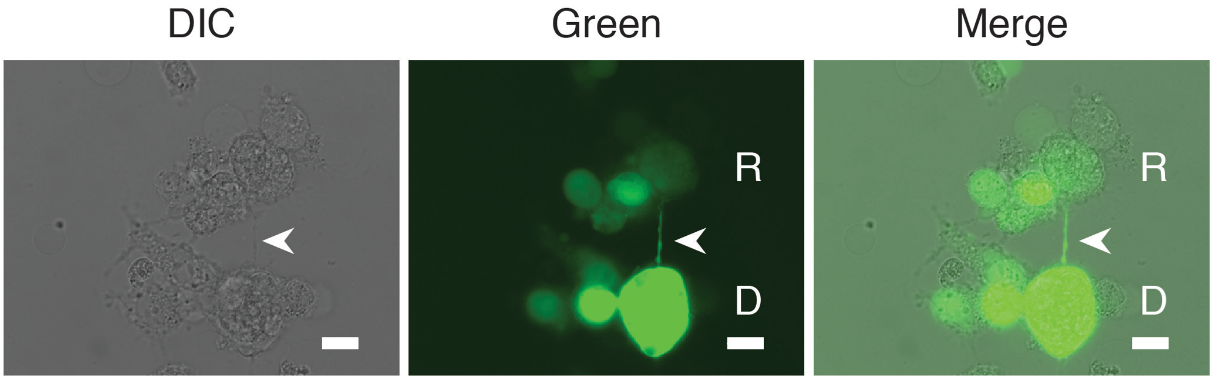

Figure S3: Intercellular transfer of NV1066-expressed eGFP between MSTO mesothelioma cells. DIC and fluorescence images were taken using inverted microscopy at 48 hours following infection; the third image is an overlay produced by merging the first two images. The initiating ("donor") cells is labeled "D"; the target ("recipient") cells is labeled "R". Scale bars: 20 μm.

Supplementary Movie Legends

Movie S1. Time-lapse imaging capturing transfer of eGFP from a NV1066 virus-infected JMN mesothelioma cell to a non-infected cell after de novo TNT formation.

Movie S2. Additional time-lapse evidence of TNT formation between JMN cells infected or not infected with eGFP-expressing NV1066 virus. Note the conformation of the TNT and its length >75 μm.

Movie S3. Time-lapse microscopic imaging of VAMT (sarcomatoid mesothelioma) cells infected with NV1066 and forming multiple TNTs to surrounding cells. VAMT cells were seeded on sterile cover glass slides and cultured in low-serum, hyperglycemic medium for five days before infection with NV1066 at an MOI of 0.1. Confocal images were taken 18 hours after transfection using a Leica Inverted microscope.

Movie S4. MSTO-211H cells transfected with NV1066 form long-range connections via TNTs, which mediate intercellular transfer of virus-expressed eGFP. Cells were cultured in low serum, hyperglycemic medium for 48 hours. NV1066 HSV was added at MOI of 0.1. Time lapse images were captured every 10 seconds using a Zeiss LSM5 Live microscope.
